# Radar Interferometry for Monitoring the Vibration Characteristics of Buildings and Civil Structures: Recent Case Studies in Spain

**DOI:** 10.3390/s17040669

**Published:** 2017-03-24

**Authors:** Guido Luzi, Michele Crosetto, Enric Fernández

**Affiliations:** Centre Tecnòlogic de Telecomunicaciòns de Catalunya (CTTC/CERCA), Geomatics Division, Avinguda Gauss, 7, E-08860 Castelldefels (Barcelona), Spain; mcrosetto@cttc.cat (M.C.); enric.fernandez@cttc.cat (E.F.)

**Keywords:** radar, interferometry, civil structures, vibration, monitoring

## Abstract

The potential of a coherent microwave sensor to monitor the vibration characteristics of civil structures has been investigated in the past decade, and successful case studies have been published by different research teams. This remote sensing technique is based on the interferometric processing of real aperture radar acquisitions. Its capability to estimate, simultaneously and remotely, the displacement of different parts of the investigated structures, with high accuracy and repeatability, is its main advantage with respect to conventional sensors. A considerable amount of literature on this technique is available, including various case studies aimed at testing the ambient vibration of bridges, buildings, and towers. In the last years, this technique has been used in Spain for civil structures monitoring. In this paper, three examples of such case studies are described: the monitoring of the suspended bridge crossing the Ebro River at Amposta, the communications tower of Collserola in Barcelona, and an urban building located in Vilafranca del Penedès, a small town close to Barcelona. This paper summarizes the main outcomes of these case studies, underlining the advantages and limitations of the sensors currently available, and concluding with the possible improvements expected from the next generation of sensors.

## 1. Introduction

The availability of microwave spaceborne remote sensing sensors aboard polar satellites has widely encouraged the development of a variety of applications of terrestrial radar sensors. These latter ones have adopted the same satellite-based techniques to monitor smaller areas, notably increasing the temporal sampling capability. In particular, synthetic aperture radar acquisitions [1] and radar interferometry [2] encouraged the development of portable systems, demonstrating the potential of microwave interferometry for the monitoring of slow deformation phenomena such as landslides, mines and glaciers [3], but also of fast phenomena as the vibration of civil structures, on which this paper is focused. Although the first pioneering experiments using coherent radar to detect displacements of civil structures date back to the 1990s [4], instruments suitable for an operational use were not available until the end of the first decade of 2000. After preliminary experiments based on the use of laboratory prototypes [5,6], commercial systems were developed [7,8]. The use of radar interferometers to measure the dynamic response of a variety of structures, such as bridges [9,10,11,12], buildings [13,14], wind farms [15], chimneys [16], ancient towers [17], and guyed masts [18] is viable and operative. The use of radar interferometers for civil structure monitoring mainly initiated in Europe, especially in Italy [19], and some experimental campaigns have been carried out in Spain. These campaigns, mainly based on the use of the commercial real aperture radar interferometric sensor IBIS-S, manufactured by IDS (Ingegneria Dei Sistemi S.p.A.), Pisa, Italy, aimed at measuring the dynamic response of different types of structures. Here we recall and discuss three examples: a suspended bridge crossing the Ebro River where it flows through Amposta [20], the Collserola Tower located in Barcelona [21], and an urban building. Radar interferometry provides sub-millimetre precision in displacement measurements from distances up to hundreds of meters, independently of the weather conditions and without any contact with the monitored structure. By contrast, the use of contact sensors sometimes requires a hazardous and costly setup. The main limitation of this technique is that it can only estimate the radar line-of-sight (LOS) component of the actual vectorial displacement, as is detailed in the next section. The campaigns confirmed the potential of this technique and exposed the weaknesses of the available sensors that could be improved by designing new advanced sensors. Although the majority of the published results are based on data collected by the same type of sensor, which is described in Section 2.2, recent articles have proposed advanced sensors. These developments aim at improving the sensor performances, but also proposing new instrumentation with reduced costs or focused on single applications. 

The paper is organized as follows: (i) a preliminary part dedicated to introduce the technique and the sensor used; (ii) a description of three experimental campaigns, underlining the main outcomes and applications; (iii) a discussion of the advantages and limitations of the sensors currently available; and, finally, (iv) a conclusion with some comments about the possible improvements expected from the next generation of sensors.

## 2. Radar Interferometry: Working Principle and Used Sensors

### 2.1. Radar Interferometry

The meaning of the radar acronym, RAdio Detection And Ranging, refers to the ability of a radar to detect and range objects using electromagnetic waves. The radar transmits microwave signals and receives echoes from the different targets contained within its antenna field of view (FOV). A radar observation uses the time elapsed between the transmission and reception of the transmitted signal waveform to provide the radar-target distance: it is a time of flight device. The basic output of a radar measurement is a signal where amplitude peaks located at different distances correspond to echo intensities from parts of the observed structure. A conventional radar, only capable of measuring the amplitude of the received signal, is (in the best case) able to detect two separated targets only if their ranges differ in the order of meters: this prevents the use of conventional radar to measure mechanical vibration of civil structures, when millimetre to micrometric displacement amplitudes are expected. Interferometry is a well-known technique, which allows retrieving the variation of wave signals’ propagation paths in the order of fractions of the operating wavelength. Considering microwave signals, and in particular wavelengths of a few centimetres (bands from X to Ku, i.e., operating electromagnetic frequencies from 8 GHz to 18 GHz), sub-millimetre variations of the range can be detected exploiting interferometric techniques. Radar interferometry allows detecting vibrations, i.e., displacement variation along time, with an accuracy of up to tens of microns in the best-case scenario, if the radar sensor, operating as a coherent detector, provides the differential phase of the received signal, namely the interferometric phase. This methodology allows retrieving, simultaneously, the displacement vibration history of different parts of the monitored structure remotely, without the installation of any artificial target.

**Figure 1:**
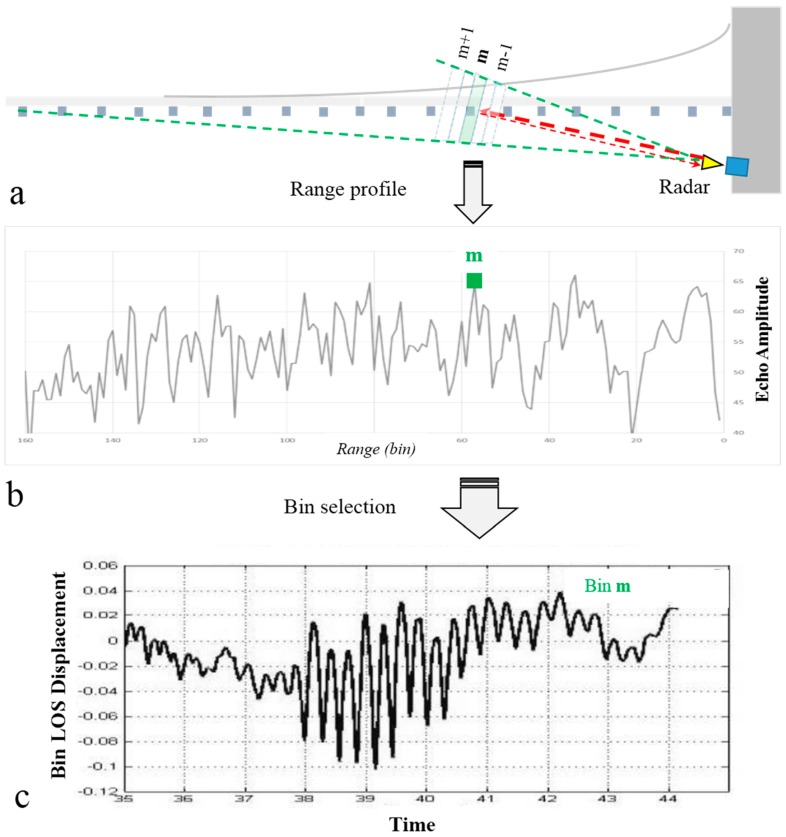
Scheme of the procedure aimed at retrieving displacement time series of a structure (the example concerns a deck bridge) from an interferometric radar acquisition. (**a**) The radar sends a signal (red arrow) and receives the backscattered energy from different parts of the structure, allowing collecting an amplitude profile; (**b**) A range profile, amplitude of the radar echoes as a function of the range, sampled at regular spatial steps is acquired; (**c**) A bin (m) is chosen; the interferometric phase of the radar echo from the selected bin is transformed in a displacement time signal using Equation (1).

A radar provides the ranging of all the targets included in its FOV, and whose backscattered signal is detectable, i.e., higher than the noise of the instrument. Two or more targets can be separated if the distance between them is larger than the radar range resolution; in the case at hand, the range resolution, dictated by the bandwidth of the radar of the microwave transceiver, is 0.5 m. Considering a case study where the radar illuminates an extended object, such as a building or a bridge, the range resolution determines the capability to sample unambiguously different parts of the monitored structure as separated elements. A regular sampling of the monitored structure is obtained by acquiring a range profile and the corresponding elemental space unit is called radar bin. A range profile is the plot of the amplitude of the received radar signal as a function of the distance, which can be expressed in radar bins or equivalent distance by multiplying the rank of the bin and the range resolution (distance = Nbin × 0.5 m in the reported experimental campaigns); outstanding peaks indicate the best reflecting parts of the structure. Figure 1a depicts the example of the location of a generic bin m. 

It is possible to obtain the displacement history of each of the separated elements; see the example shown in Figure 1. This involves three main steps. The first is collecting a range profile, sampled at regular spatial steps. Secondly, we can associate the interferometric phase of the related echo to parts of the structure when the intensity of the radar echo coming from the separated elements assures an adequate signal-to-noise ratio (SNR). Finally, a displacement history is retrieved by transforming the temporal variations of the interferometric phase of a radar bin using the following simple Equation (1):

(1) $$d_{LOS}(t)=\frac{\lambda }{4\pi }\cdot \Delta \phi (t)$$

where Δϕ(t) is the difference between the phases measured in two consecutive radar acquisitions and *λ* the wavelength of the transmitted wave.

The retrieval of the LOS displacement from the measured differential phase of the received radar signal is possible because a coherent radar also provides the phase value of the reflected signal which allows, through interferometry, evaluating range variations in terms of fractions of a wavelength of the propagating radar wave. When the displacement ranges are within ±*λ*/4, the phase change is linearly related to the variation of distance *d_LOS_*(*t*) occurring between two successive radar acquisitions, as stated by Equation (1). Considering that *λ*/4 is larger than 4 mm in the case at hand, and that the expected structure displacements range from a millimetre to tens of microns, this approximation usually works and unwrapping procedures are simple or even not necessary [22]. According to Equation (1), the shorter the wavelength, the higher the sensitivity of the measurement in terms of displacement. For a Ku radar, with a wavelength *λ* = 1.74 cm (operating frequency = 17.2 GHz), a phase variation of 1°, typically achievable using a state-of-the-art sensor, corresponds to a displacement of approximately 20 microns: the expected displacement precision at best measuring conditions (high SNR), is of the order of tens of microns. The main limitations of this technique is that, considering a three-dimensional displacement ***d***(*t*), the proposed technique can only estimate the projection of ***d***(*t*) in the LOS d_LOS_; if ***d***(*t*) is perpendicular to the LOS, the displacement vector ***d***(*t*) cannot be observed by the radar. As far as the precision of the measurement is concerned, this is related to the SNR which, at a first approximation, can be estimated through the ratio between the strength of the radar echo from the selected bin and the instrumental noise (thermal), and theoretically calculated using Equation (2). The SNR of the acquisition mainly depends on the transmitted power, the thermal noise of the receiver, the distance, geometric factors (shape and orientation of the target), and dielectric characteristics of the target. A discussion about some of these issues can be found in [23,24,25].

(2) $$\sigma_{displ}=\frac{\lambda }{4\pi }\frac{1}{\sqrt{SNR}}$$

In actual cases, the SNR must be statistically estimated as in [23] due to the presence of secondary undesirable vibrating targets (clutter) included in the radar bin. For this reason, and due to the high variability of the experimental conditions, the estimate of an adequate SNR is usually carried out through data analysis and interpretation. 

### 2.2. The Used Sensor

The Centre Tecnològic de Telecomunicacions de Catalunya (CTTC) owns a commercial radar with interferometric capability: the IBIS-S manufactured by IDS. The system consists of a sensor module, a control PC, a power supply unit, and data processing software. The coherence and, hence, the interferometric capability of this radar, is obtained using a step frequency continuous wave transceiver, which transmits an electromagnetic signal at a central frequency of 17.2 GHz (Ku band) with a maximum bandwidth of 300 MHz, corresponding to a range resolution of 0.5 m. Range resolution is intended here as the minimum distance required between two targets to be separated, i.e., seen as two different subsequent bins. This radar operates in a different way with respect to the conventional pulsed radar, using a frequency modulation instead of an amplitude modulation, i.e., sending pulses with increasing frequency, spanning a finite bandwidth, usually referred to as a synthetic pulse. This technical feature allows performing high phase stability and SNR at the same time, without differing, from a physical point of view, from the signal of a conventional pulsed radar. 

The radar instrument is mounted on a tripod equipped with a rotating head to adjust the bearing of the sensor towards the investigated structure. The main sensor characteristics are summarized in Table 1. The maximum acquisition rate is 200 Hz, depending on the selected maximum range and decreasing as the maximum operating distance increases. Details on the radar equipment can be found in [23]. The location and dimension of the measured radar bins are dictated by the transmitting and receiving antennas FOV and by the range resolution. The bins that correspond to the highest signal peaks are usually selected to analyse their displacement time series. The sensor unit is managed by a control PC, through standard USB communication, which is provided with system management software used to configure the acquisition parameters, store the measured data and show the displacements in real time. The power supply unit provides about 5 h of autonomy. The equipment is quick and easy to install and can be used both day and night, and in almost any weather conditions. The majority of published papers about the use of radar sensor for vibration monitoring over this topic is based on the use of this system, but in the last years novel sensors have been proposed by different researchers; a brief recall of some of these papers will be provided in the discussion section.

## 3. The Experimental Campaigns

Three case studies, where the radar sensor described in the previous section has been used to study the dynamical behaviour of three civil structures, are reported in this section. For each case, we focus on the outstanding results: for a detailed description, readers are addressed to the original papers recalled here. Among the several campaigns carried out by CTTC team in the last decade, we select the following cases to underline the reliability of the technique in different measuring conditions and to demonstrate the high precision obtainable with optimum measuring conditions. Displacements ranging from millimetres to tens of microns are detected from operating distances ranging from tens to hundreds of meters, without any contact or artificial targets mounted on the investigated structure. Bridge monitoring is one of the applications most reported in literature [19], while building monitoring represents a challenging case due to the reduced amplitude of vibration. From an operational point of view, the effectiveness of the application has been consolidated in several studies, showing how the estimated displacement can be profitably used to estimate the modal response of the structures, which is a topic of great concern in structure health monitoring [26].

### 3.1. The Amposta Cable-Suspended Bridge

#### 3.1.1. Description of the Structure

**Figure 2:**
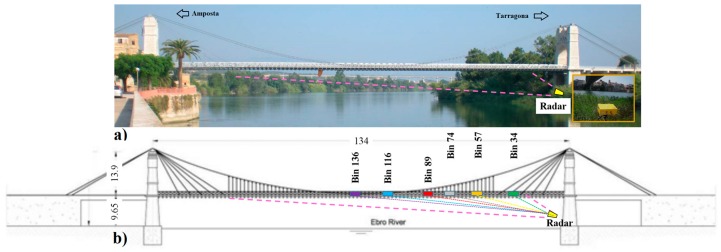
(**a**) Photograph of the Amposta bridge roughly indicating (pink dashed lines) the area illuminated by the radar during the acquisitions aimed at detecting the displacements of the deck. A photograph of the radar sensor taken during data acquisition is shown in the yellow box located at the bottom right of the figure. (**b**) Plan of the bridge showing the radar bins selected for Figure 3.

The structure is a cable-suspended bridge, which crosses the River Ebro near the town of Amposta (Catalonia). It consists of a main span of 134.0 m and two side-spans of 46.90 m each. The main suspension system consists of two series of four suspension cables, which run along the right and left side of the bridge. The main cables, approximately 240 m long, cover the entire structure, passing over the piers through a saddle, and are anchored to the original massive concrete anchorage blocks. Sixty-nine equally-spaced hangers on each side support the central part of the deck, whereas five inclined stay cables on each side support the deck in the parts close to the towers. There are stone masonry towers on both sides of the bridge hosting the saddles over which the main cables pass. Figure 2 shows a photograph of the bridge with the radar position. After its construction in 1921, the bridge experienced a dramatic demolishment, a re-construction, and recently underwent two important strengthening interventions, carried out in 1972 and 2007 [27]. Its monitoring is of great interest for maintenance purposes and the use of remote techniques is suitable considering the high costs and operational difficulties of a standard survey. An exhaustive description of the entire analysis of the bridge monitoring is reported in [20]. Interferometric processing of radar acquisitions using output-only identification techniques [28,29] allowed estimating deck dynamic deflections and retrieving the vibration modes of the deck.

#### 3.1.2. Results

The dynamic response of the bridge and of all the arrays of stay cables were investigated in operational conditions using an Ibis-S system on 23 and 24 July 2013. The microwave interferometer was installed in two different acquisition geometries. Here, we focus on the first one, with the radar sensor positioned at the base of one tower, under the bridge as depicted in Figure 2; this location aimed at detecting the displacements of the deck. The LOS estimates obtained from radar data interpretation are used to retrieve the deflections of the deck. Further positions, on both sides at the level of the bridge deck, not discussed here, were used in order to obtain a complete study of the dynamical response of each array of the suspension cables and the inclined stay cables [18]. 

**Figure 3:**
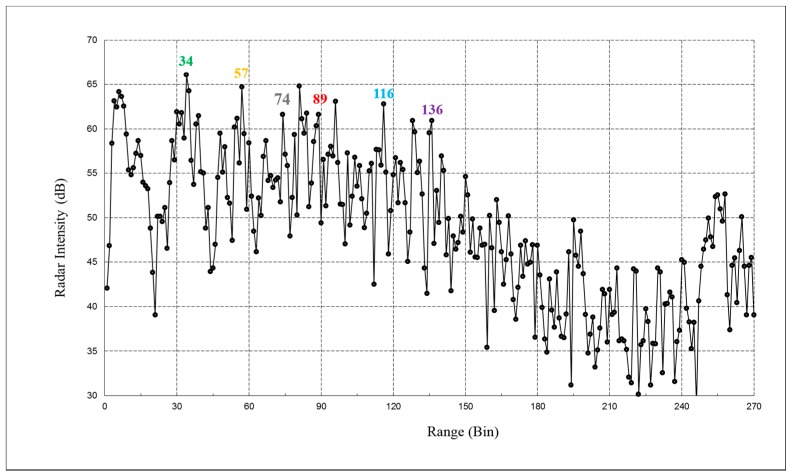
Range profile of the Amposta bridge. Each radar bin corresponds to 0.5 m of range resolution. Numbers indicate the radar bins selected for estimating the deck deflections. The position of the bins is indicated in Figure 2b.

**Figure 4:**
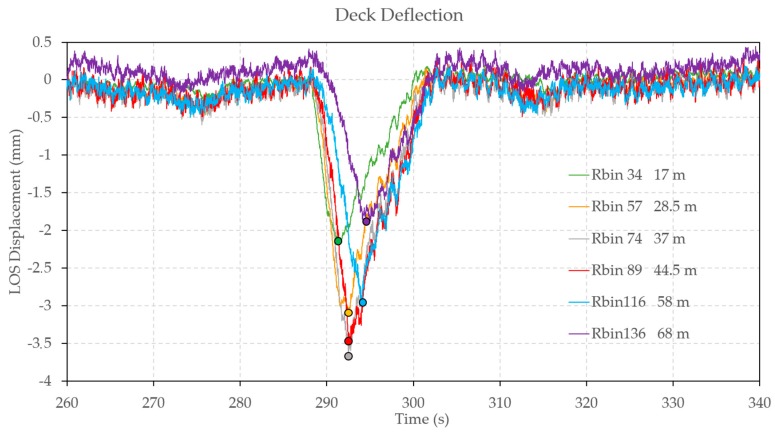
LOS displacement time histories of the bridge deck retrieved from radar measurements, associated to the passage of a truck, at different distances from the Tarragona-side tower. Black rounded points indicate the minimum value. In the legend are reported the ranges, expressed as bin number and meters.

The operational test was carried out with the excitation mainly provided by the road traffic on the bridge during acquisition. After acquiring a range profile of the deck bridge, shown in Figure 3, we selected a set of radar bins with the best SNR. The location of the selected bins is depicted in Figure 2. Vibrations were attained by processing their corresponding temporal samples; the total duration of the acquisition was 3600 s sampled at a rate of 100 Hz. The high sensitivity of the measurement can be appreciated by observing the radar response of the bridge deck associated to the passage of a truck: a cut of the time histories of some bins at different distances from the tower is depicted in Figure 4.

The analysis of this plot indicates that the maximum deflection (minimum value of displacement) occurs at the centre of the bridge (bin 136), as expected from the deflection induced by the fundamental mode. The spatio-temporal pattern of the maximum deflections, indicated by the circles in Figure 4, is the result of the motion of truck along the bridge. 

A straightforward operational use of these data is the calculation of the first modal shapes of the bridge deck using an output-only identification technique (frequency domain decomposition, FDD [29], which can be implemented using the commercial software ARTeMIS^®^, Structural Vibration Solutions A/S, Aalborg East, Denmark [30]. The frequencies associated to each mode can also be obtained calculating the power spectral density (PSD) of the selected bins using the Welch method [31]. 

**Figure 5:**
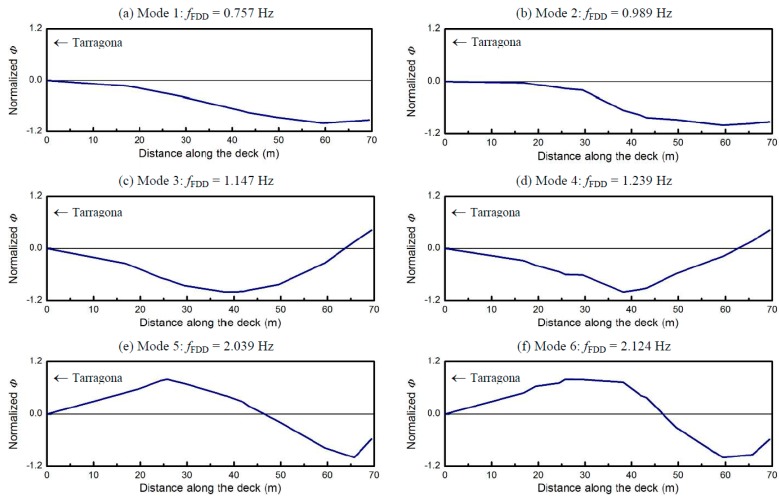
Vibration modes of the bridge deck identified using FDD technique from the deflections measured on the bridge deck. Reprinted from [20].

The modal shapes obtained for the Amposta bridge deck radar monitoring are depicted in Figure 5 (reprinted from [20]). Although the radar sampled the entire bridge, we only draw half of the deck length. We focused on this part, closer to the sensor location, due to the higher SNR of the concerned bins assuring the best measurement precision. The regular and smooth shapes of the curves shown in Figure 5 confirmed the good quality of the displacement data provided by the microwave interferometer.

### 3.2. The Collserola Tower

#### 3.2.1. Description of the Structure

**Figure 6:**
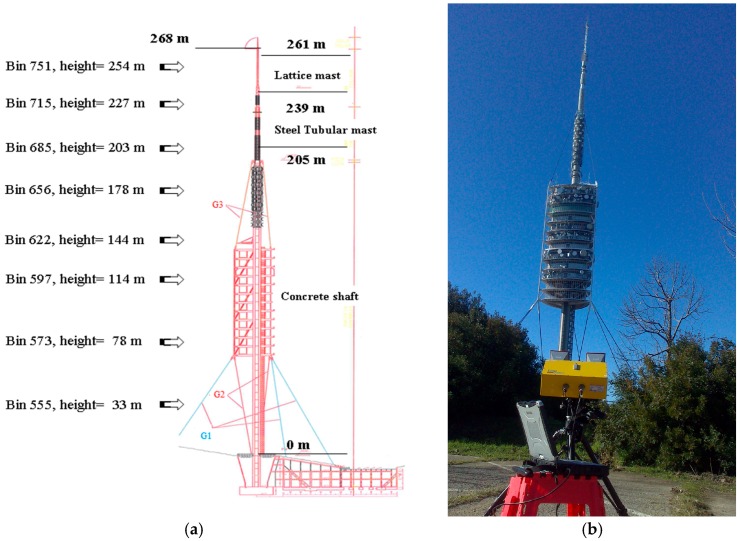
(**a**) Scheme of the main parts of the tower indicating the height of the analysed radar bins. Reprinted from [21]; and (**b**) photograph of the Collserola Tower and showing the radar installation.

The Collserola Tower, designed by the architect Norman Foster and built for the 1992 Olympic Games [32], is one of the outstanding structures of Barcelona. It serves as a telecommunications infrastructure for the city and surrounding area. It is 288 m tall and, despite the strong and complex structure of anchoring, usually undergoes wind forcing with oscillations of significant amplitudes. The structure (Figure 6a) is composed of five main elements: a concrete shaft, a metal mast, a block of platforms, nine pre-tensioned steel guys and three fibre guys, anchoring the tower to concrete blocks in the ground. The lower guys are steel cables (composed of a different number of strands), whereas the remaining ones are Kevlar fibre guys. There are several reasons that suggested testing the use of a remote approach for its monitoring. First, its size: an exhaustive sampling using a conventional contact technique would require a very large number of sensors distributed along the structure. Secondly, the presence of a number of antennas with strong electromagnetic interference disturbances makes the installation of conventional sensors, such as accelerometers, unadvisable due to the strong electromagnetic noise, which can totally compromise their functioning. Finally, radar monitoring can be performed remotely, avoiding an interruption of its service for safety regulations. The radar equipment was installed at different distances from the tower base, up to several hundreds of meters, during the dynamic tests. Despite the complexity of the illuminated surface, the radar was able to extract the general dynamical behaviour of the tower providing the main modal shapes. Figure 6b shows a photograph of the tower taken from the radar position.

#### 3.2.2. Results

**Figure 7:**
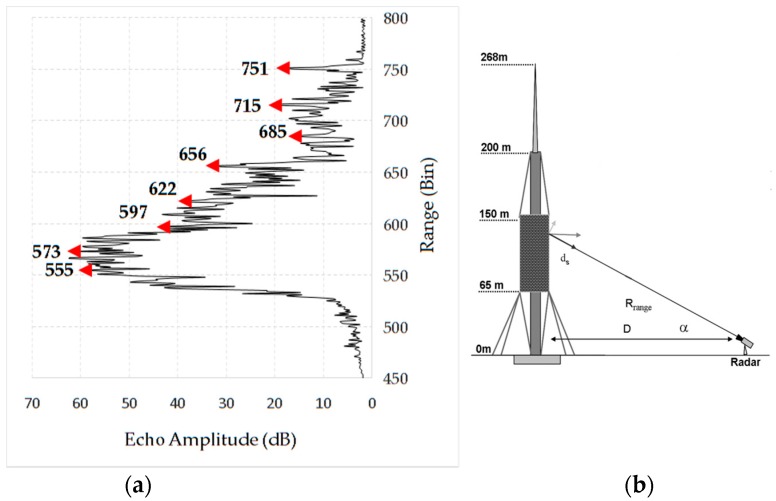
(**a**) Range profile indicating the bins corresponding to the peak, depicted in Figure 6a. and (**b**) scheme of the tower and acquisition geometry indicating the LOS component, *d_S_*, measured by the radar. D: distance radar-tower, R: radar range; α: inclination angle.

**Figure 8:**
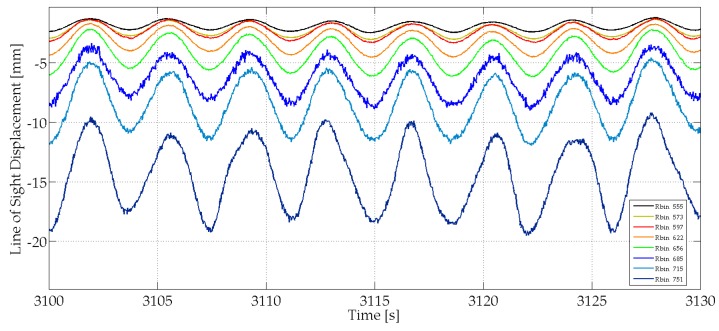
Zoom of the time series of the LOS displacement retrieved from radar data for some selected bins indicated by different colours. The periodicity of all the samples and the increase of the amplitude with the height of the bin, as expected for the fundamental oscillation mode of the tower, are evident.

**Figure 9:**
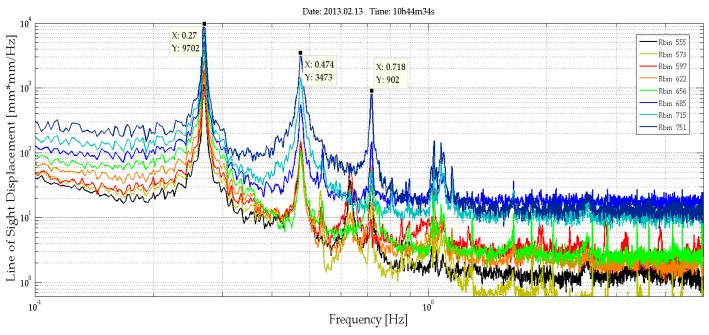
PSD (amplitude of LOS displacement) calculated for the samples depicted in Figure 8. Markers indicate the intensity in mm^2^/Hz and the frequency in Hz of the peaks. The three main peaks at 0.27 Hz, 0.474 Hz, and 0.718 Hz, identified as the frequencies of the first oscillation modes of the tower, are common to all of the bins. Secondary peaks are associable to the contribution of the cables.

A set of bins corresponding to parts of the tower at different heights (see Figure 6a,b) is analysed with the aim of obtaining an exhaustive sampling of the entire structure. Figure 7 shows the range profile indicating the bins that correspond to the peak selected for the study and a scheme of the tower and the acquisition geometry. The radar signal was sampled at 40 Hz. The analysis of a 30-s cut of the acquired LOS displacement signal shown as a function of time displays a clear harmonic behaviour for all the bins (Figure 8), with the amplitude decreasing with height, as expected for the first mode vibration of this kind of vertical structure. The spectral analysis of the entire samples showed the presence of different peaks. Figure 9 shows the PSD calculated for eight bins using the Welch method, with 66% of overlapping sub-samples, and windowed by a Hanning function. The temporal extension of the samples is 3600 s, a lapse which is approximately 1000 times larger than the previously-measured period of vibration (T = 3.6 s), which provides a statistically robust sampling of the stationarity of the signal of interest. 

It is worth noting that, despite the low SNR of the signal corresponding to the higher part of the tower, i.e., those with bin number bigger than 597, the radar is capable to precisely estimate these displacement histories because their amplitudes are larger than the accuracy of the instrument.

**Figure 10:**
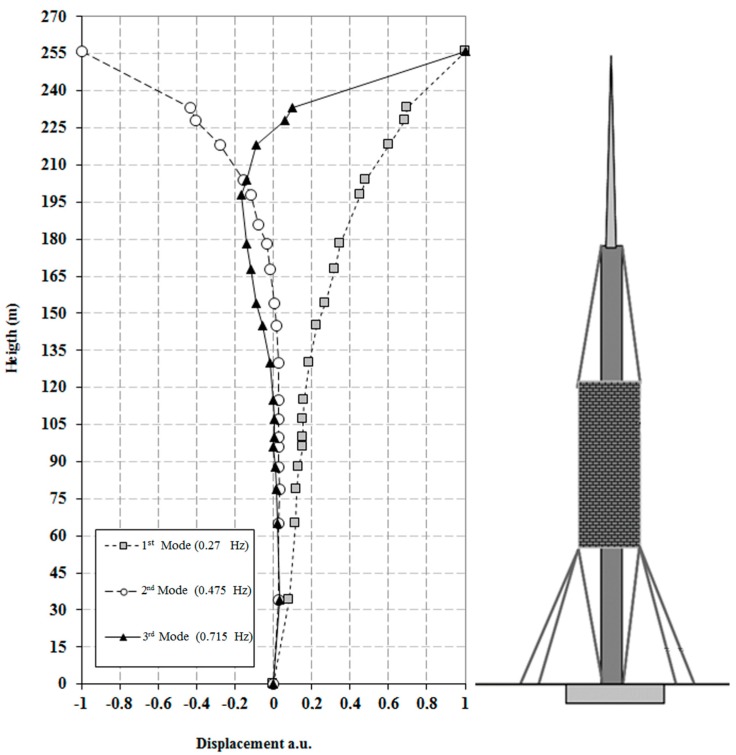
Plot of the first vibration modes identified using the FDD technique Reprinted from [21].

The presence of three main peaks at 0.27 Hz, 0.474 Hz, and 0.718 Hz is a common feature for all of the bins. The 0.27 Hz is the strongest signal and is a plausible value for the tower’s first mode; the other two outstanding peaks are present considering a larger set of bins (twenty), used for retrieving the displacements occurring along the structures and estimating its global deformation. Figure 10 shows the three first modal shapes. The secondary minor peaks present in Figure 10 can be interpreted by combining these data with some acquisitions obtained by directly monitoring the cables. The radar acquisitions are able to offer an overall, simultaneous view of such a tall structure.

### 3.3. An Urban Building

#### 3.3.1. Description of the Structure

One of the outstanding performances of the use of a radar interferometer to monitor civil structures is the capability to provide a high sensitivity and precision for displacement measurements [33]. The technique can be considered highly reliable, especially with respect to optical sensors whose use can often be jeopardized by atmospheric propagation and the limited observation geometries available to provide a high SNR despite their unique sensitivity. The amplitude of vibration of a typical urban building, i.e., with a regular shape and no more than 10 stories, is of the order of tens of microns and this value is close to the best accuracy of the radar sensor reported here. In this section, we describe the acquisition of radar data aimed at detecting the vibration frequency of an urban building located at Vilafranca del Penedès (Barcelona), a ten-storey reinforced concrete structure. 

#### 3.3.2. Results

**Figure 11:**
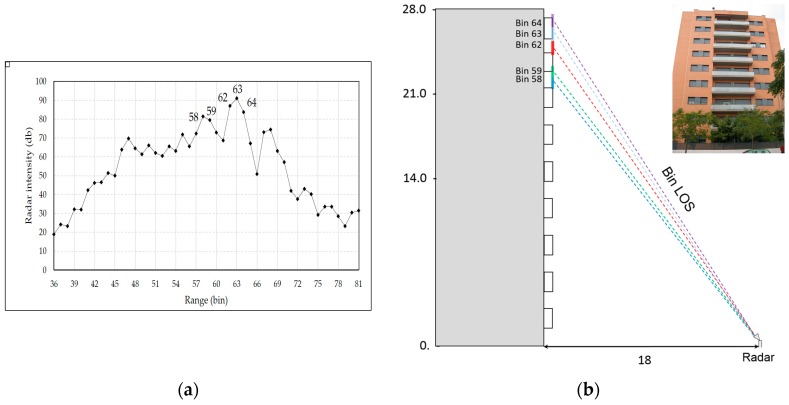
(**a**) Range profile acquired illuminating the façade of a nine-storey building indicating the bins selected for retrieving the displacement. (**b**) Scheme of the radar acquisition indicating the location of the analysed radar bins. A photograph of the monitored building located in Vilafranca del Penedès (Barcelona) is also shown. Distance and heights are in meters.

**Figure 12:**
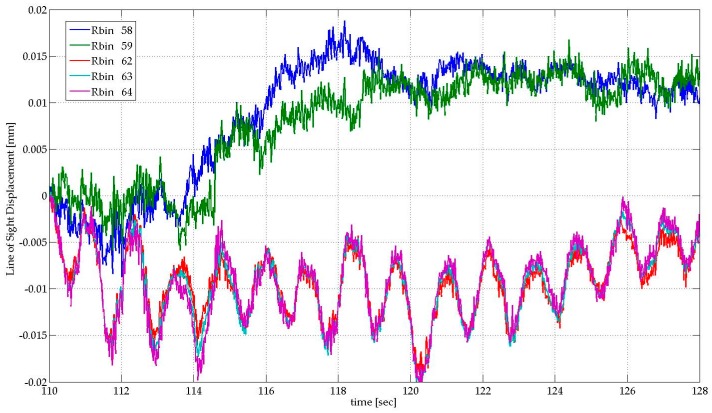
LOS displacement time series retrieved from radar data for the bins showed in Figure 11a. The bins with higher SNR (62, 63, and 64) show a clear periodicity with respect to bins 58 and 59, which have a lower SNR (see Figure 11a).

**Figure 13:**
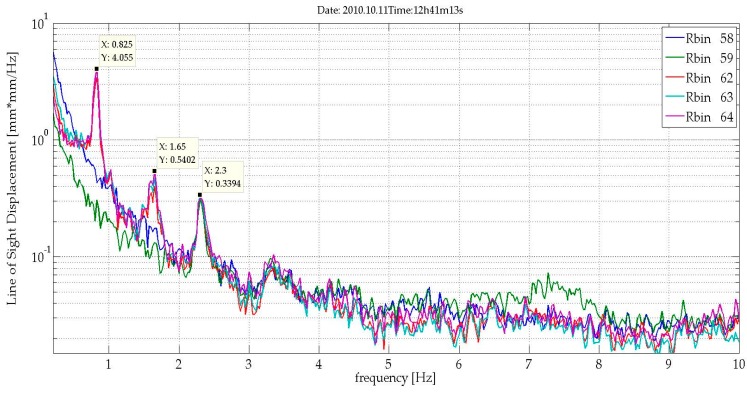
PSD calculated using the Welch method for the five selected bins and a signal duration of 5 min.

Figure 11a shows the range profile obtained at a distance of approximately ten meters from the building façade. The main peaks, whose SNR is high enough to assure a fine precision in displacement estimation, can be associated to the discontinuity wall-terrace, which act as a corner reflector. Five bins (see Figure 11a) with SNR greater than 80 dB were selected to guarantee measuring conditions assuring the best precision, according to Equation (2). We evaluated the displacement behaviour of the building and obtained its main vibration frequencies. Figure 12 shows a LOS displacement time series of 18 s corresponding to the five bins selected. The clear periodicity observed for bins 62, 63, and 64, whose SNR is greater than 84 dB, indicates a vibration frequency of 0.82 Hz (period of about 1.22 s); the corresponding amplitude is about 10 microns. The calculation of the PSD of this stationary signal was applied to accurately assess the spectral characteristics of the acquired signal. Figure 13 shows the PSD calculated for a total duration of five minutes. The presence of a clear harmonic component at 0.82 Hz (the corresponding period is 1.22 s) can be observed for bins 62, 63, and 64. By contrast, bins 58 and 59 do not show the same behaviour; the signal is probably too small because the displacement is below the noise threshold at the corresponding height of the building. This example suggests that to estimate sub-millimetre displacements for buildings with similar height than the one considered in this case study, also in agreement with Equation (2), a SNR value greater than 80 dB can be a good reference. This parameter is strongly linked to the geometrical and dielectric characteristics of the backscattering surface [34]. For this reason, when low displacement amplitudes are expected, the observation position of the radar must be optimized experimentally case by case. When this condition is not satisfied, and adequate SNR values are not available, there is still the possibility to install radar reflectors in the surface of the analysed structure. However, the use of reflectors, which are required in the case of demanding high accuracy (see for example [7]), reduce one of the main advantages of the proposed technique, i.e., the capability to measure completely remotely without any direct access to the structure. As far as the frequency value obtained through the spectral analysis is concerned, this can be reasonably associated to the first vibrating mode of the building. This parameter, which is related to the mass and stiffness of the structure, is usually estimated using simple empirical formulas, although these parameters are affected by several factors, like the height of the building, and based on assumptions concerning the shape and structural characteristics of the building [35]. Considering, for example, the empirical approach which states that the oscillating frequency F_0_ can be estimated as F_0_ = 10/(number of stories), the obtained value of 0.82 Hz is a bit low; considering the possible presence of a further storey in the basement, which would result in F_0_ = 0.91 Hz, the error decreases to less than 10%. A second, weaker, peak associable to a second harmonic of F_0,_ and finally a peak, which is common to all of the bins, probably associable to a spurious, systematic vibration, affecting the mechanical stand of the radar, is appreciable in Figure 13. This assumption is supported by the fact that the frequency is present for all of the bins and with the same shape and amplitude. Due to the lack of detailed information about the building structural characteristics, and the challenging measuring condition, a robust assessment of the first vibration frequency demands a validation from conventional sensors. 

## 4. Discussion and Conclusions

The case studies described here, which represent three examples of application of radar interferometry carried out in Spain, confirmed the main performances of a radar interferometer for vibration monitoring of civil structures and the potential of the acquired data as the input for modal analysis. The technique demonstrated to be a valuable remote tool under different situations, i.e., with different targets and measuring distances. This is probably the main benefit of the proposed approach. However, a limitation persists, i.e., this technique only provides a component of the displacement; however, this is imputable to the interferometric technique and can be overridden using combinations of more sensors. An example of the use of a set of sensors to provide 3D displacement estimates have been investigated in [36]. At the same time, the results of the described experimental campaigns also show some limitations of the available system, suggesting possible improvements of at least two performances of the system: the range resolution and the accuracy in displacement detection. 

The best range resolution, taking the Ibis-S system as reference, is limited to 0.5–0.75 m (mainly depending on the regulations on spectrum management and frequency allocation). The actual resolution worsens when observing with a high incidence angle, as in the building monitoring, due to the projection of the LOS direction. The range resolution must be improved to allow a more spatially-detailed description of the structures at hand. Concerning the accuracy, the amplitude of displacements of these kinds of structures is very close to the system accuracy, as observed in the urban building monitoring discussed in this paper. This issue reduces the number of useful radar bins and an adequate modal shape retrieval based on these data can be difficult. A solution for these two drawbacks is the use of radar sensors working at a higher frequency, i.e., shorter wavelength. In this case, wider radiofrequency bandwidths are available, which can increase both the sensitivity and the accuracy of the phase measurement. A higher SNR is achievable using antennas with higher gain without compromising the handiness of the sensor for their small size. 

Some authors recently performed preliminary tests of novel sensors working at a higher frequency and with a larger radio frequency bandwidth able to provide an improved range resolution [37]. Some prototypes of new systems profit from low-cost transceivers available off-the-shelf. The proposed system uses an off-the-shelf linear frequency modulated K-band (centre frequency: 24 GHz) sensor, with a lower maximum operating range with respect to the commercial interferometer, but whose development costs are drastically reduced. In some cases, imaging capabilities at higher frequencies have been investigated [38]. In other cases, sensors based on the use of innovative techniques as “noise radar” has been proposed [39], or radar systems devoted to specific applications have been described [40]. Another approach is the one proposed in [41]; here the authors report on the development and test of a handy, user-friendly, moderate-cost system; a simple measurement procedure is developed to provide highly-portable equipment. In this last case, the sensor operates in the X band, a lower frequency with respect to the Ibis-S system. Simpler sensors have also been proposed, as in [42]. 

In conclusion, after almost two decades of relative stagnation with respect to the development of new sensors, a promising research activity aiming at improving the performances of the technique discussed in this article is in progress. 

## Figures and Tables

**Table 1:** Main characteristics of the radar system.

Radar Main Characteristics	
Operating frequency	17.2 GHz
Maximum distance	1000 m
Maximum range resolution	0.5 m
Maximum sampling rate	200 Hz
Nominal displacement accuracy	2 × 10^−5^ m
